# Posterior mesh inguinal hernia repairs: a propensity score matched analysis of laparoscopic and robotic versus open approaches

**DOI:** 10.1007/s10029-022-02680-0

**Published:** 2022-09-20

**Authors:** M. Reinhorn, N. Fullington, D. Agarwal, M. A. Olson, L. Ott, A. Canavan, B. Pate, M. Hubertus, A. Urquiza, B. Poulose, J. Warren

**Affiliations:** 1 Boston Hernia, 20 Walnut Street, Suite 100, Wellesley, MA 02481 USA; 2 Mass General Brigham - Newton-Wellesley Hospital, Newton, MA USA; 3 Department of Surgery, Massachusetts General Hospital, Boston, MA USA; 4 Department of Population Health Sciences, Weill Cornell Medicine, New York, NY USA; 5 Center for Abdominal Core Health, Department of Surgery, The Ohio State University Wexner Medical Center, Columbus, OH USA; 6 Department of Surgery, Division of Minimal Access, and Bariatric Surgery, Prisma Health Upstate, 701 Grove Rd, ST 3, Greenville, SC 29605 USA

**Keywords:** TREPP, Open preperitoneal inguinal hernia repair, OPP, Posterior mesh inguinal hernia repair, MIS inguinal hernia repair

## Abstract

**Purpose:**

International guidelines suggest the use of lapro-endoscopic technique for primary unilateral inguinal hernia (IHR) because of lower postoperative pain and reduction in chronic pain. It is unclear if the primary benefit is due to the minimally invasive approach, the posterior mesh position or both. Further research evaluating posterior mesh placement using open preperitoneal techniques is recommended. A potential benefit of open preperitoneal repair is the avoidance of general anesthesia, as these repairs can be performed under local anesthesia. This study compares clinical and patient-reported outcomes after unilateral laparo-endoscopic, robotic, and open posterior mesh IHRs.

**Methods:**

We performed a propensity score matched analysis of patients undergoing IHR between 2012 and 2021 in the Abdominal Core Health Quality Collaborative registry. 10,409 patients underwent a unilateral IHR via a posterior approach. Hernia repairs were performed via minimally invasive surgery (MIS) which includes laparoscopic and robotic transabdominal preperitoneal (TAPP), laparoscopic totally extraperitoneal (TEP), or open transrectus preperitoneal/open preperitoneal (TREPP/OPP) approaches. Propensity score matching (PSM) utilizing nearest neighbor matching accounted for differences in baseline characteristics and possible confounding variables between groups. We matched 816 patients in the MIS cohort with 816 patients in the TREPP/OPP group. Outcomes included patient reported quality of life, hernia recurrence, and postoperative opioid use.

**Results:**

Improvement was seen after TREPP/OPP as compared to MIS IHR in EuraHS at 30 days (Median(IQR) 7.0 (2.0–16.64) vs 10 (2.0–24.0); OR 0.69 [0.55–0.85]; *p* = 0.001) and 6 months (1.0 (0.0–4.0) vs 2.0 (0.0–4.0); OR 0.63 [0.46–85]; *p* = 0.002), patient-reported opioid use at 30-day follow-up (18% vs 45% OR 0.26 [0.19–0.35]; *p* < 0.001), and rates of surgical site occurrences (0.8% vs 4.9% OR 0.16 [0.06–0.35]; *p* < 0.001). There were no differences in EuraHS scores and recurrences at 1 year.

**Conclusions:**

This study demonstrates a potential benefit of open posterior mesh placement over MIS repair in short-term quality of life and seroma formation with equivalent rates of hernia recurrence. Further study is needed to better understand these differences and determine the reproducibility of these findings outside of high-volume specialty centers.

**Supplementary Information:**

The online version contains supplementary material available at 10.1007/s10029-022-02680-0.

## Introduction

Inguinal hernia repair (IHR) is performed in over 20 million individuals annually worldwide [1]. Considerable debate remains over the optimal surgical approach for unilateral inguinal hernia repair. International guidelines suggest that in skilled hands, posterior laparo-endoscopic mesh repair may lead to reduced early postoperative pain and reduced incidence of chronic groin pain [2–4], while maintaining equivalent rates of recurrence [3, 5]. Posterior mesh repair can be accomplished using a minimally invasive (laparoscopic and/or robotic) or an open approach [6–8]. Minimally invasive (MIS) IHR is further divided into transabdominal preperitoneal (TAPP) and totally extraperitoneal (TEP) approaches. Several open posterior approaches exist that also allow preperitoneal mesh placement, including the Transrectus Preperitoneal repair (TREPP), Transinguinal Preperitoneal repair (TRIPP), Open Preperitoneal repair (OPP), and the Kugel repair. However, there is currently insufficient comparative data to make any conclusive recommendations. As such, further evaluation of open techniques for posterior mesh placement with comparative analysis to currently accepted minimally invasive repair is warranted.

The disadvantage of open posterior repairs is the potential for violation of both the anterior and posterior planes. However, the Transrectus Preperitoneal (TREPP), Open Preperitoneal (OPP), and the Kugel repair all involve incisions above the traditional open anterior inguinal hernia repair, thus sparing the anterior plane while still allowing for posterior mesh placement. One key differentiator between open posterior and MIS IHR is choice of anesthetic. To perform an MIS IHR, general anesthesia with muscle relaxation is typically required to allow for pneumoperitoneum and placement of the mesh. By contrast, open posterior mesh repairs can often be performed utilizing local anesthesia with intravenous sedation, which improves short-term outcomes [4, 9]. Studies evaluating TREPP/OPP have thus far been limited, but some single-institution observational studies have shown promising outcomes [10–13]. Comparisons of TREPP to MIS TEP and open Lichtenstein approaches demonstrate no significant differences in recurrence rates, postoperative complications, or postoperative pain [5, 14], and a recent meta-analysis on TREPP demonstrated low risk of recurrence, chronic pain, hematoma, and wound infection [15]. While evaluation of the quality of hernia repair has traditionally involved comparison of recurrence rates, patients themselves also measure the success of their surgery by their quality of life in recovery. Patient reported outcomes such as post-operative pain, ability to return to work and exercise, and aesthetic outcomes play a significant role in determining the quality of repair [16]. The purpose of this study is to compare postoperative clinical and patient-reported outcomes in individuals who underwent unilateral MIS IHR compared to open posterior mesh IHR. We hypothesized that open preperitoneal repairs have similar outcomes to MIS repairs and may be a good option for patients who may benefit from avoidance of general anesthesia.

## Methods

### Data collection

The Abdominal Core Health Quality Collaborative (ACHQC) is a national registry that collects short- and long-term hernia-specific data, including patient reported outcomes related to hernia repairs with the goal of improving surgical quality and value [17]. We utilized data collected in the ACHQC to compare MIS versus open posterior mesh repairs of unilateral inguinal hernia. Between August 2012 and December 2021, 25,975 patients underwent inguinal hernia repair. Patients who underwent bilateral inguinal hernia repair, anterior approaches, transinguinal posterior approaches, combined inguinal and ventral hernia repair, or repair of multi recurrent inguinal hernias were excluded (those with first time recurrence were included). 10,409 patients underwent unilateral IHR via a posterior approach; 908 patients underwent TREPP and 9501 MIS (4,073 rTAPP; 3448 TEP; 1980 TAPP). We then matched 816 patients in the MIS cohort with 816 patients in the TREPP/OPP group for our analysis. Outcomes included patient reported quality of life, hernia recurrence, and postoperative opioid use. Table 1 highlights the factors that were matched between the two cohorts, while Table 2 highlights the differences in surgeon volume, choice of anesthesia and mesh fixation. This study was approved by the Institutional Review Board at Prisma Health Upstate.

**Table 1:** Standardized mean differences (SMDs) in baseline characteristics in the MIS and Open IHR cohorts after propensity score matching

	MIS posterior (TAPP/TEP)	Open posterior (TREPP)	SMD
*N*	816	816	
Age capped at 90 (mean (SD))	59.58 (13.63)	59.66 (14.15)	0.006
Male (%)	767 (94.0)	770 (94.4)	0.016
*Race/ethnicity (%)*			0.093
White, not of Hispanic origin	781 (95.7)	767 (94.0)	
Black, not of Hispanic origin	2 (0.2)	6 (0.7)	
Hispanic	10 (1.2)	14 (1.7)	
Other	23 (2.8)	29 (3.6)	
*BMI capped 15–60 (mean (SD))*	26.07 (4.10)	26.03 (4.10)	0.011
*Insurance*			0.059
Private	575 (70.5)	553 (67.8)	
Medicare	217 (26.6)	236 (28.9)	
Other	24 (2.9)	27 (3.3)	
*ASA class (%)*			0.051
1	220 (27.0)	202 (24.8)	
2	514 (63.0)	527 (64.6)	
3	82 (10.0)	87 (10.7)	
4 +	0 ( 0.0)	0 (0.0)	
Hypertension (%)	219 (26.8)	233 (28.6)	0.038
Diabetes mellitus (%)	19 ( 2.3)	28 (3.4)	0.066
Chronic obstructive pulmonary disease (%)	3 ( 0.4)	5 (0.6)	0.035
Anti-platelet medications (%)	85 (10.4)	80 (9.8)	0.02
Anti-coagulation medications (%)	9 ( 1.1)	12 (1.5)	0.033
Smoker within one year (%)	33 ( 4.0)	32 (3.9)	0.006
Indication for surgery: enlarging hernia	24(2.9)	21(2.6)	.022
Indication for surgery: painful bulge	806(98.8)	807(98.9)	.011
Indication for surgery: recurrent hernia	75(9.2)	62(7.6)	.057
Prior pelvic operation	31(3.8)	39(4.8)	.048
Prior mesh	26(3.2)	18(2.2)	.061
*Medial type hernia size (%)*			0.039
No Hernia	579 (71.1)	579 (71.0)	
I (< 1.5 cm or < 1 fingertip)	30 ( 3.7)	26 (3.2)	
II (1.5-3 cm or 1–2 fingertips)	170 (20.9)	170 (20.9)	
III (> 3 cm or > 2 fingertips)	35 ( 4.3)	40 (4.9)	
*Lateral type hernia size (%)*			0.044
No Hernia	185 (22.7)	198 (24.3)	
I (< 1.5 cm or < 1 fingertip)	82 (10.0)	78 (9.6)	
II (1.5-3 cm or 1–2 fingertips)	493 (60.4)	488 (59.9)	
III (> 3 cm or > 2 fingertips)	56 ( 6.9)	51 ( 6.3)	
Scrotal component (%)	29 ( 3.6)	29 ( 3.6)	< 0.001
Patient or surgeon reported opioid use in last 30 days at time of operation	4(0.6)	7(0.9)	.093
Other substance use (non-opioid) (%)	183 (31.4)	288 (35.8)	0.093
EuraHS overall score at baseline (mean (SD))	26.60 (17.92)	25.10 (18.21)	0.083

**Table 2:** Unilateral inguinal hernia surgeon volume, anesthesia and mesh fixation characteristics

	MIS	TREPP/OPP	*P* value
Number of surgeons	130	9	
Surgeon volume range since ACHQC inception	1–1028	1–646	
Surgeon volume median (IQR)	40 (17–78)	1 (1–106)	
Yearly volume range	0–174	0–207	
Yearly volume median (IQR)	12 (6–19)	0.4 (0.2–112)	
*Anesthesia type*	*N* = 816	*N* = 816	
General	813/818 (99.63%)	228/816 (27.94%)	*p* < 0.001
Sedation	2/816 (0.25%)	589/816 (72.18%)	*p* < 0.001
TAPP block	68/816 (8.33%)	3/816 (0.37%)	*p* < 0.001
Intraoperative local anesthetic	138/816 (16.91%)	793/816 (97.18)	*p* < 0.001
Spinal	0/816 (0.00%)	1/816 (0.12%)	*p* = .317
*Fixation type*	*N* = 816	*N* = 816	
Tacks	333/816 (40.81%)	1/816 (0.12%)	*p* < 0.001
Adhesives	59/816 (7.23%)	8/816 (0.98%)	*p* < 0.001
Staples	29/816 (3.55%)	0/816 (0.00%)	*p* < 0.001
Sutures	237/816 (29.04%)	776/816 (95.10%)	*p* < 0.001

### Surgical procedure

**Fig. 1:**
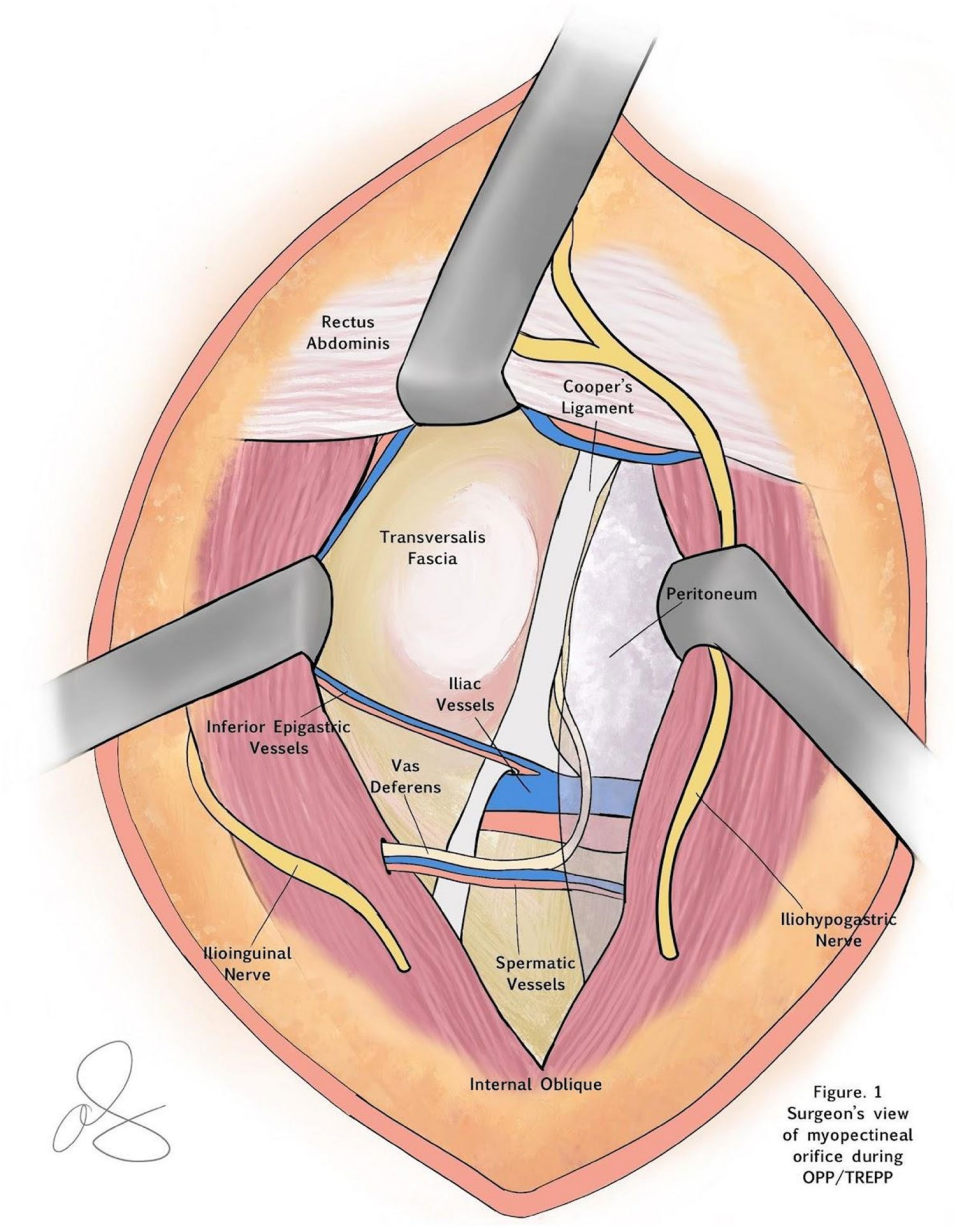
Representation of the myopectineal orifice as seen by a surgeon over time through a 4 cm left lower abdominal incision. A TREPP is performed through a 4-5 cm lower abdominal incision. Using a headlight, the surgeon can visualize the entire myopectineal orifice in its entirety, by having assistants retract the abdominal wall in different directions

**Fig. 2:**
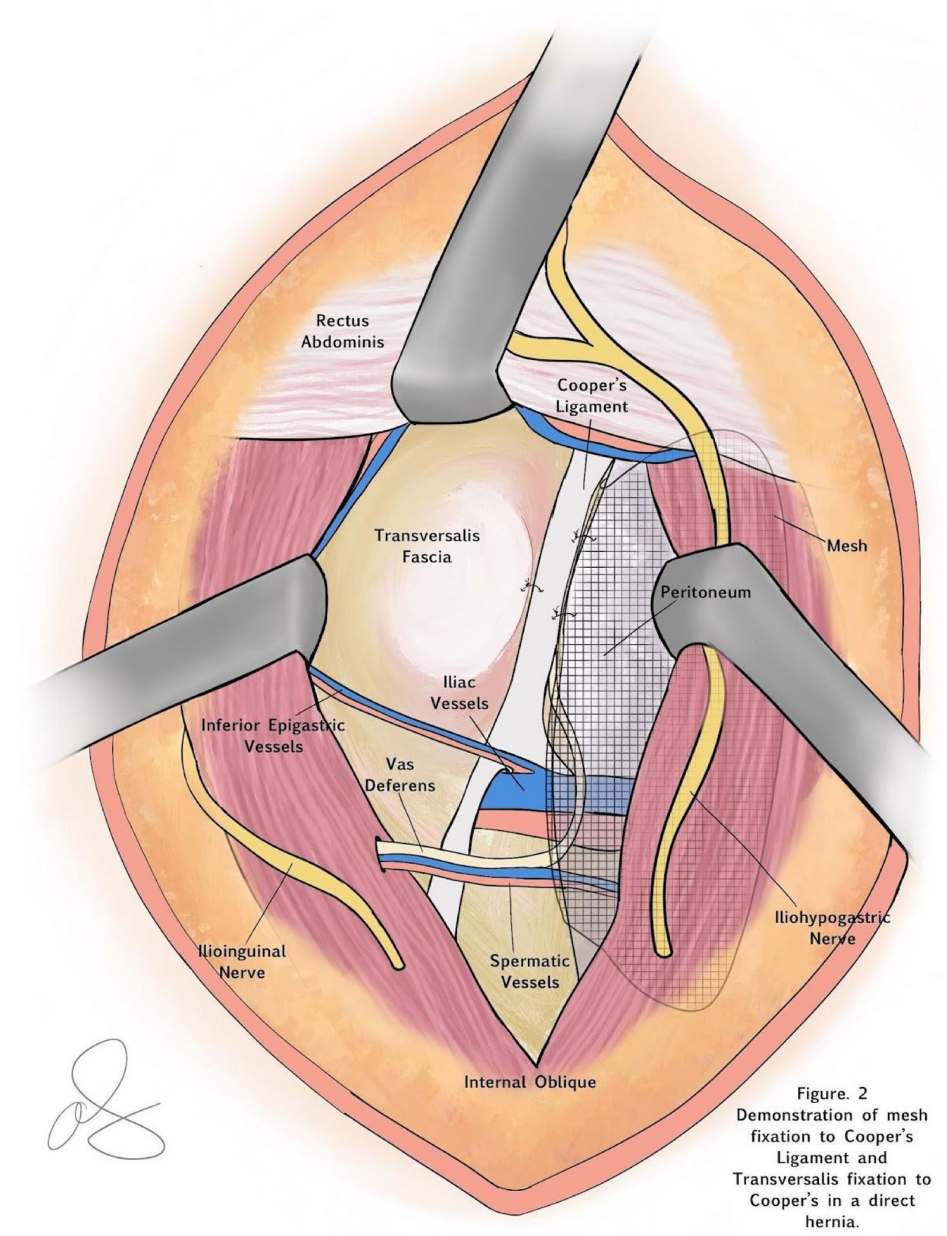
Completed dissection and mesh placement, including suture fixation

For analytic purposes, laparoscopic TAPP, robotic TAPP (rTAPP), and laparoscopic TEP repairs are grouped as MIS IHR based on the clinical and technical similarities between these operations. MIS approaches have been well described in the literature with similar outcomes across techniques [18–36]. To simplify the nomenclature, in the ACHQC, posterior mesh approaches that do not include a transinguinal dissection were grouped under TREPP. These include OPP, TREPP and Kugel. These approaches involve a lower abdominal incision and opening of the external oblique aponeurosis superior to the inguinal canal. The iliohypogastric nerve is identified and the internal obliques are separated just superior to the nerve (OPP/Kugel) or incising the rectus sheath and retracting the rectus medially at the same level (TREPP), (Fig. 1). This dissection avoids dissection in the interparietal plane between the external and internal obliques where anterior repair is typically performed, thus minimizing scarring in the inguinal canal and allowing unobstructed anterior repair in the event of recurrence requiring future anterior repair. Open dissection of the preperitoneal space is performed and in most patients, a 14 cm by 11 cm mesh is placed into the preperitoneal space, covering the myopectineal orifice of Fruchaud. Mesh is fixed to Cooper's ligament with suture. In medial defects, excess transversalis fascia is inverted and sutured to Cooper's ligament as well [37, 38], (Fig. 2). The technique for these procedures has been described in detail elsewhere [10, 39–49]. The open posterior repairs will be referred to as TREPP for simplicity.

### Outcomes

Data collected include patient demographics and comorbidity, surgical details, clinical outcomes, and patient reported outcomes (PRO) before, during, and after unilateral IHR procedures as described previously [50]. The primary outcome is patient reported quality of life using the EuraHS scores at 30-day, 6-month, and 1-year after surgery. The EuraHS is a validated quality of life measurement tool for inguinal hernia. The tool assesses pain (range 0–30), restriction of activity (range 0–40), and cosmetic discomfort (range 0–20) due to the hernia or from surgery [51] with total scores ranging from 0 to 90, A lower score signifies an improved QOL.

Secondary outcomes include composite hernia recurrence, perioperative complications, surgical site occurrence or infection, and opioid use. Clinical or radiographic recurrence is recorded by the clinician at any point after surgery. Patient surveys are filled out in the office or sent to ACHQC patients at 30 days, 6 months and then once per year after surgery. Patient reported outcome recurrence are recorded at or after the 1-year mark. Composite recurrence is defined by the Hernia Recurrence Inventory which includes physical exam or radiographic imaging at any point post-operatively or a patient reported bulge at the site of the hernia at a 1 year or greater after hernia repair.

### Statistical methods

Patient-level, hernia, and operative characteristics were compared between individuals who received TREPP and MIS IHR. Pearson’s chi-squared and Wilcoxon rank sum tests were used to conduct bivariate tests comparing categorical and continuous covariates, respectively. Time-to-recurrence was examined using Kaplan–Meier recurrence free estimation and log-rank test to compare recurrence curves between operative approaches. An advantage of evaluating recurrence as time-to-event is the ability to use all information available to compute 1-year recurrence probability, including endpoint and censoring information, and to account for varying length of follow up. Because there is much more statistical information, survival analyses tend to have greater statistical power to detect effects over methods for binary outcomes [52, 53]. Although the time it might take after an IHR for the hernia to recur is not possible to predict definitively in the clinical setting, our Kaplan–Meier analysis is statistically precise and its estimate of recurrence-free probability is an unbiased representation of the true time-to-event data as reflected by the relatively stable width of the curve's confidence intervals up to 3 years. This method to characterize long-term recurrence has been established in statistical literature and in hernia recurrence reporting [54, 55]. Additional pairwise analysis was performed to detect differences in TREPP and each MIS IHR technique.

To address selection bias and systematic differences in baseline covariates, we created a propensity-score matched cohort. A logistic regression model was used to estimate the propensity score for operative approach conditional on covariates identified a priori. Covariates included in the propensity score model were age, gender, race, BMI, insurance status, ASA class, comorbidities, indication for surgery (enlarging hernia, painful bulge, recurrent hernia), prior pelvic operation, prior mesh, hernia size, scrotal component, history of substance use, history of opioid use, behavioral health history, and EuraHS quality of life score measured at baseline. A 1:1 nearest-neighbor matching algorithm with a caliper of 0.2 was used to match TREPP with MIS IHRs [56, 57]. Balance was assessed by examining the standardized mean differences (SMD) of baseline covariates where a SMD < 0.1 was considered good balance. This resulted in 816 TREPP and 816 MIS IHR patients for comparison. Odds and hazard ratios and their 95% confidence intervals were estimated using logistic, proportional odds, or Cox proportional hazards models for binary, patient-reported, and time-to-event outcomes, respectively. To assess the difference in EuraHS quality of life scores between surgical approaches for populations with the same baseline score, we adjust for baseline scores in a proportional odds regression model.

To address missingness in baseline data, we performed 30 iterations of multiple imputation using a predictive mean matching algorithm to preserve baseline information. Results were pooled using the across approach [58]. Planned propensity score matched subgroup analyses were performed to compare TREPP to each type of MIS approach. Lastly, to address possible bias due to informative missingness in the outcomes, we performed a tipping point analysis as a sensitivity analysis.

## Results

### Primary outcome

#### Patient reported quality of life

Postoperative quality of life (QOL) is the primary outcome examined in this work. In the matched analysis, after accounting for baseline scores, there was a significantly better (lower) EuraHS QOL score in TREPP compared to MIS at the 30-day (Median(IQR) 7.0 (2.0–16.64) vs 10 (2.0–24.0); OR 0.69 [0.55–0.85]; *p* = 0.001) and 6 months (1.0 (0–4.0) vs 2.0 (0.0–4.0); OR 0.63 [0.46–0.85]; *p* = 0.002) time points. These differences disappeared by one-year (1.0 (0.0–6.0) vs 1.0 (0.0–4.0); OR 0.82 [0.54–1.23]; *p* = 0.347) (Table 3). Additionally, domain-specific subanalysis performed post-hoc revealed lower pain domain scores after TREPP repair at 30 days, but not at 180 days, in pairwise comparisons between each matched TREPP cohort to corresponding laparoscopic TAPP, TEP, and robotic TAPP cohorts (Supplemental Tables 1, 2, 3).

**Table 3:** MIS vs open propensity score matched outcomes

Adjusted outcome analysis	MIS posterior (TAPP/TEP)	Open posterior (TREPP)	*P* value	OR	95% CI
	(*N* = 816)	(*N* = 816)			
*EuraHS QoL score from 30-day survey (score 0–90)*			0.001	0.686	(0.552, 0.853)
*N*	481	515			
Median (interquartile range)	10.00 (2.00—24.00)	7.00 (2.00—16.64)			
*EuraHS QoL score from 6-month survey (score 0–90)*			0.002	0.625	(0.461, 0.847)
*N*	238	326			
Median (interquartile range)	2.00 (0.00—8.00)	1.00 (0.00—4.00)			
*EuraHS QoL score from 1-year survey (score 0–90)*			0.347	0.817	(0.537, 1.246)
*N*	121	198			
Median (interquartile range)	1.00 (0.00—6.00)	1.00 (0.00—4.00)			
*Patient reported opioid use in last 30-days at 30-day follow-up*			< 0.001	0.261	(0.192, 0.352)
0	227/408 (55.64)	391/475 (82.32)			
1–4	106/408 (25.98)	63/475 (13.26)			
5–10	55/408 (13.48)	17/475 (3.58)			
11 or more	20/408 (4.90)	4/475 (0.84)			
30-day surgical site infection (SSI)	0/719 (0.00)	1/757 (0.13)	0.995	N/A	N/A
*30-day surgical site occurrence (SSO-EI)*	35/719 (4.87)	6/757 (0.79)	< 0.001	0.156	(0.059, 0.347)
Nonhealing incisional wound	1/35 (2.86)	0/6 (0.00)			
Wound serous drainage	1/35 (2.86)	1/6 (16.67)			
Stitch abscess	0/35 (0.00)	1/6 (16.67)			
Seroma	29/35 (82.86)	3/6 (50.00)			
Hematoma	6/35 (17.14)	2/6 (33.33)			
30-Day SSO or SSI requiring procedural intervention (SSOPI)	1/719 (0.14)	1/757 (0.13)	0.971	0.95	(0.038, 24.047)
30-Day any NSQIP complications	15/719 (2.09)	7/757 (0.92)	0.073	0.438	(0.166, 1.045)
Bowel obstruction	1/719 (0.14)	0/757 (0.00)			
PE	0/719 (0.00)	1/757 (0.13)			
UTI	1/719 (0.14)	0/757 (0.00)			
*Urinary retention requiring catheter placement*	10/717 (1.39)	3/757 (0.40)			

### Secondary outcomes

#### Recurrence rates

**Fig. 3:**
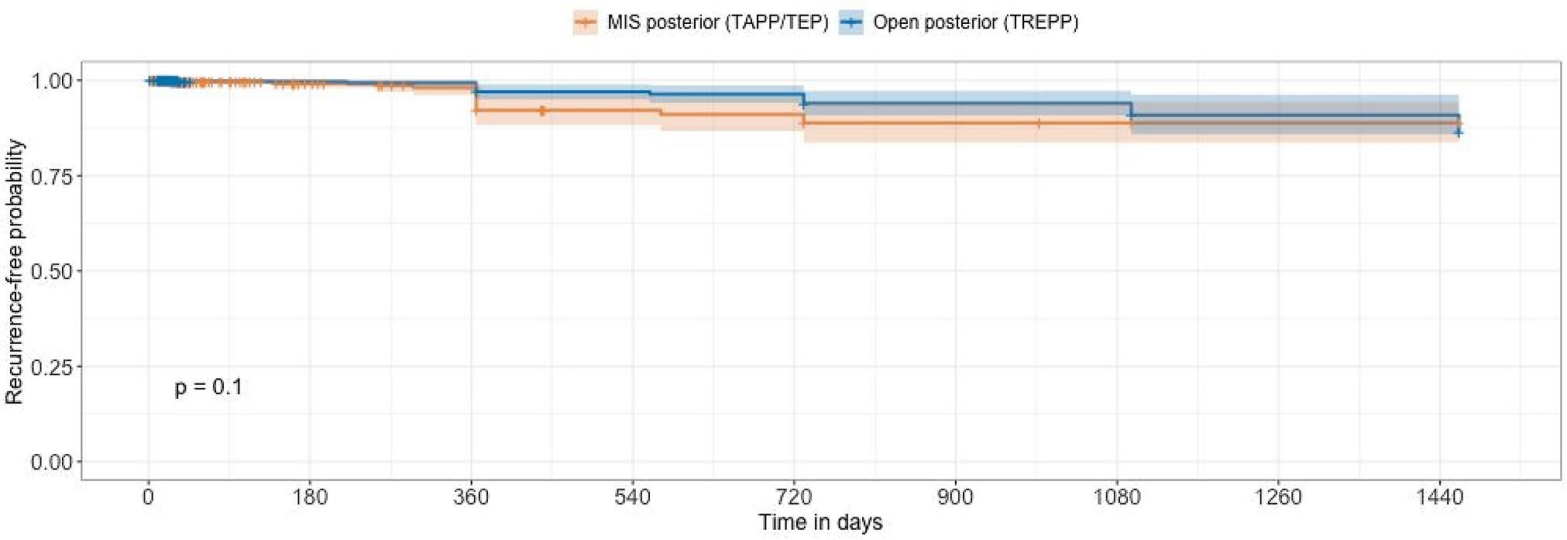
Recurrence free probability of MIS vs TREPP repairs

At 1 year, 2/296 composite recurrences were reported in the TREPP group and 4/174 composite recurrences reported in the MIS group. The Kaplan–Meier time-to-event log-rank test did not reveal a statistically significant difference in hernia recurrence risk between the TREPP and MIS repair cohorts (Fig. 3). Subgroup analysis using Kaplan–Meier recurrence free estimation and log-rank test as previously described showed minimal differences in the composite recurrence risk between the TREPP, TAPP, TEP and rTAPP groups (Supplemental Fig. 1).

#### Postoperative opioid use

Postoperative opioid use at 30-day follow-up was significantly lower in the TREPP cohort (OR 0.26 [0.19–0.35]; *p* < 0.001) (Table 3). Further, in the matched comparison between TREPP and each of the MIS subgroups, significantly lower opioid use was still evident in TREPP. Specifically, at 30-day follow-up, matched analyses demonstrated that those undergoing TREPP were more likely to not use any opioids postoperatively versus those undergoing MIS repairs (TREPP 255/315, 81% versus TAPP 125/254, 49% *P* < 0.001; TREPP 258/314, 82% versus TEP 131/293, 45% *P* < 0.001; TREPP 314/383, 82% versus rTAPP 174/308, 56% *P* < 0.001).

#### Safety, peri-operative aspects, and adverse events

The 30-day frequency of surgical site occurrences (SSOs) was 4.9% (35/719) for the MIS repair cohort compared to 0.8% (6/757) in the TREPP cohort (OR 0.16 [0.06–0.35]; *p* < 0.001). The majority of SSOs in all groups were seromas. There were no statistically significant differences in 30-day SSOs or surgical site infections requiring procedural intervention, rates of postoperative bleeding, peripheral nerve injury, postoperative respiratory failure, pulmonary embolism, ileus, bowel obstruction, DVT, or UTI between the TREPP and MIS repair cohorts (Table 3). No statistical differences were seen between TREPP and MIS IHR in urinary retention; however, reduced rates of urinary retention were appreciable in TREPP versus TAPP (TREPP 0.5% (3/561); TAPP 2.0% (11/558) *P* = 0.11) and TREPP versus rTAPP (TREPP 0.5% (3/649); rTAPP 1.9% (12/632) *P* = 0.17).

## Discussion

In this analysis comparing TREPP and MIS approaches to posterior unilateral inguinal hernia repair, TREPP was associated with decreased postoperative pain domain score, lower opioid use and better overall quality of life compared to MIS approaches at 1 month and 6 months after surgery. No statistically significant differences between the repairs were noted in QOL or recurrence at 1 year after surgery. These results suggest that an open posterior mesh IHR utilizing local anesthesia and sedation may have short-term benefits over the more common MIS repairs.

A key difference between the TREPP and MIS repairs is the ability to perform TREPP with local anesthesia and sedation while avoiding general anesthetic and muscle relaxation. Reduced postoperative pain, early mobilization after surgery, and reduced cost associated with local anesthetic use in IHR has been demonstrated in prior studies [4, 10, 59–62], and our study confirms this is true for open posterior mesh repairs as well. Additionally, general anesthetic is independently associated with higher healthcare cost compared to local anesthetic [60].

The improvement in the PRO pain domain and differential opioid consumption seen in this study is likely multifactorial. Peritoneal entry, incision, and closure used for TAPP and robotic TAPP repairs may account for the increased pain, particularly as PRO in the pain domain was less pronounced between TREPP and TEP repairs. Additionally, suture fixation was performed in 96% of the open repairs while tacks were used in 42% of MIS approaches. There are mixed data on the relationship between mesh fixation and postoperative pain, thus obfuscating any clear conclusions on the impact of this difference on our observed outcomes. We also found lower rates of postoperative seroma in the TREPP group. While most seromas are self-limited with minimal clinical significance, the impact on quality of life is unclear and may be a contributing factor to lower QOL scores in the MIS group. Several meta-analyses report that seroma formation rates are higher following MIS compared to open repairs [63–66]. Studies have shown reduction in seroma formation after MIS repair when transversalis fascia is inverted to close the dead space [37, 38]. This technical detail is not captured in the ACHQC and we cannot determine its direct impact on reported outcomes. However, the high single-surgeon volume of TREPP in which this maneuver is routinely performed suggests some correlation with transversalis fascia fixation and decreasing seroma rate. It is also important to point out that, though statistically significant differences in PRO were found, the clinical significance of this finding is not defined.

Decreased postoperative opioid use after TREPP can at least partly be attributed to administration of local anesthesia. However, selection bias is a significant factor here due to practice-specific prescribing patterns and focus on opioid reduction. Better patient education on anticipatory management of postprocedural pain and decreased surgeon prescribing can significantly impact opioid consumption, but was not standard across patients studied [67, 68]. Still, the differences in opioid use between TREPP and MIS approaches are significant and warrant further investigation. Differential prescribing practices, patient education, surgeon experience, and patient expectations all impact opioid use. Opioid reduction initiatives within the ACHQC for the last two years have had variable success, which may lead to some of the differences seen in our study. A recent analysis of ACHQC data indicates that opioid prescribing is an independent risk factor for opioid consumption, thus implicating individual surgeon practice and management of patient expectations as a potential confounder [69]. Combined with the discrepancy in the number of participating surgeons contributing data to TREPP vs MIS, this introduces additional bias. Nonetheless, the observation of lower opioid use in TREPP suggests that significantly fewer opioids can likely be prescribed in all posterior inguinal hernia repairs and therefore reduce surgeons’ impact on the opioid epidemic in the United States.

Reported incidence of urinary retention after IHR ranges from 1 to 22% [70, 71]. The use of general anesthesia is an independent risk factor for retention [72, 73]. Patients undergoing TREPP were much more likely to avoid general anesthesia which may explain why patients undergoing TREPP had reduced rates of urinary retention over MIS repairs. Though these differences did not reach statistical significance in our analysis, the corresponding p-values and confidence intervals approached the alpha threshold of 0.05, with three- to fourfold increase consistently evident in the rate of retention following a laparoscopic or robotic repair. Given the large number of patients undergoing IHR annually, this is arguably an important observation resulting in reduced quality of life after surgery and prolonged hospital stay for those patients who develop urinary retention [60, 70–73]. Additional data collection and analysis will be required to further investigate this trend.

No statistically significant differences were seen in recurrence rates after IHR between approaches. This is not surprising given that the end result of the repairs is identical, utilizing similar size mesh for complete posterior coverage of the myopectineal orifice. However, our relatively short duration of follow-up limits any true comparison in terms of hernia recurrence. Lack of long-term follow-up is a known limitation of the ACHQC. Greater than 1-year follow-up in this study is seen in less than 25%. To account for this, we used a Kaplan–Meier disease-free model to predict recurrence based on the hernia inventory scores obtained throughout the study period. Efforts are ongoing to improve long-term follow-up in the ACHQC, for instance, by implementing an interface with Center for Medicare Services (CMS) data.

There are important limitations to this study that deserve discussion. First, a minimal clinically important difference (MCID) has not been established for the EuraHS, thus any clinical significance of our findings has yet to be established. The TREPP repairs were performed by a significantly smaller subset of surgeons compared to MIS repair, which likely introduces significant selection bias into our results. This bias is introduced by the large number of TREPP cases being performed by a small subset of high-volume surgeons. Admittedly, heterogeneity in surgeon training, learning curve, and case volume potentially influence these outcomes. In our analysis, one surgeon (first author) performed the majority of the TREPP repairs but trained an MIS surgeon (second author) on the TREPP approach during the study period. In training, we identified two learning curves to this operation and feel that they are hurdles that can be overcome with a methodical training approach. The first learning curve is to become competent at the operation, which we estimate at 25 cases. The second learning curve is to become an expert teacher of the operation, and we estimate that would require 100–200 cases with deliberate practice [74]. Since both MIS and TREPP repairs are offered in our practice, we feel that in patients who have access to a skilled surgeon, TREPP is a good alternative to MIS and can allow patients to  avoid general anesthesia.

Finally, to address missing data, tipping point sensitivity analysis was performed. This suggested that a substantial degree of differential missingness, where unobserved outcome rates/means would need to be more than 75% higher/lower than observed rates/means, would have to be present in the data to alter the conclusions of this analysis. As such, it is unlikely that missing outcome data are dramatically different from observed data.

## Conclusion

This study demonstrates a potential benefit of open posterior mesh placement over MIS repair in short-term quality of life and seroma formation with equivalent rates of hernia recurrence. Further study is needed to better understand these differences and determine the reproducibility of these findings outside of high-volume specialty centers.

## Supplementary Information

Below is the link to the electronic supplementary material.

Supplementary file1 (DOCX 63 KB)

## References

[1] Kingsnorth A, LeBlanc K (2003). Hernias: inguinal and incisional. Lancet.

[2] Bökkerink WJ, Persoon AM, Akkersdijk WL, van Laarhoven CJ, Koning GG (2017). The TREPP as alternative technique for recurrent inguinal hernia after Lichtenstein’s repair: a consecutive case series. Int J Surg.

[3] Persoon AM, Bökkerink WJV, Akkersdijk WL, van Laarhoven CJHM, Koning GG (2018). Case series of recurrent inguinal hernia after primary TREPP repair: re-TREPP seems feasible and safe. Int J Surg Case Rep.

[4] Lange JF, Lange MM, Voropai DA, van Tilburg MW, Pierie JP, Ploeg RJ (2014). Trans rectus sheath extra-peritoneal procedure (TREPP) for inguinal hernia: the first 1000 patients. World J Surg.

[5] Faessen JL, Stoot JHMB, van Vugt R (2021) Safety and efficacy in inguinal hernia repair: a retrospective study comparing TREPP, TEP and Lichtenstein (SETTLE). Hernia 10.1007/s10029-020-02361-w 10.1007/s10029-020-02361-w 33400030

[6] Koning GG, Andeweg CS, Keus F, van Tilburg MW, van Laarhoven CJ, Akkersdijk WL (2012). The transrectus sheath preperitoneal mesh repair for inguinal hernia: technique, rationale, and results of the first 50 cases. Hernia.

[7] Nyhus LM, Stevenson JKHH (1959). Preperitoneal herniorrhaphy: a preliminary report in fifty patients. West J Surg Obstet.

[8] Kugel RD (1999). Minimally invasive, non laparoscopic, preperitoneal, and sutureless, inguinal herniorrhaphy. Am J Surg.

[9] Ceriani V, Faleschini E, Bignami P, Lodi T, Roncaglia O, Osio C, Sarli D (2005). Kugel hernia repair: open "mini-invasive" technique. Personal experience on 620 patients. Hernia.

[10] Voyles CR, Hamilton BJ, Johnson WD, Kano N (2002). Meta-analysis of laparoscopic inguinal hernia trials favors open hernia repair with preperitoneal mesh prosthesis. Am J Surg.

[11] Aitola P, Airo I, Matikainen M (1998). Laparoscopic versus open preperitoneal inguinal hernia repair: a prospective randomized trial. Ann Chir Gynaecol.

[12] Johansson B, Hallerbäck B, Glise H, Anesten B, Smedberg S, Román J (1999). Laparoscopic mesh versus open preperitoneal mesh versus conventional technique for inguinal hernia repair. Ann Surg.

[13] Hamza Y, Gabr E, Hammadi H, Khalil R (2010). Four-arm randomized trial comparing laparoscopic and open hernia repairs. Int J Surg.

[14] Zwols TLR, Slagter N, Veeger NJGM, Möllers MJW, Hess DA, Jutte E (2020). Transrectus sheath pre-peritoneal (TREPP) procedure versus totally extraperitoneal (TEP) procedure and Lichtenstein technique: a propensity-score-matched analysis in Dutch high-volume regional hospitals. Hernia.

[15] Hajibandeh S, Hajibandeh S, Evans LA (2022). Meta-analysis of the outcomes of Trans Rectus Sheath Extra-Peritoneal Procedure (TREPP) for inguinal hernia. Hernia.

[16] Campanelli G (2022). Quality of life is the most important outcome measure of hernia repair. Hernia.

[17] AlMarzooqi R, Tish S, Huang LC, Prabhu A, Rosen M, Review of inguinal hernia repair techniques within the Americas Hernia Society Quality Collaborative (2019). Hernia.

[18] HerniaSurge Group (2018). International guidelines for groin hernia management. Hernia.

[19] Gass M, Banz VM, Rosella L, Adamina M, Candinas D, Güller U (2012). TAPP or TEP? Population-based analysis of prospective data on 4,552 patients undergoing endoscopic inguinal hernia repair. World J Surg.

[20] Köckerling F, Bittner R, Jacob DA, Seidelmann L, Keller T, Adolf D, Kraft BKA (2015). TEP versus TAPP: comparison of the perioperative outcome in 17,587 patients with a primary unilateral inguinal hernia. Surg Endosc.

[21] Krishna A, Misra MC, Bansal VK, Kumar S, Rajeshwari S, Chabra A (2012). Laparoscopic inguinal hernia repair: transabdominal preperitoneal (TAPP) versus totally extraperitoneal (TEP) approach: a prospective randomized controlled trial. Surg Endosc.

[22] Tetik C, Arregui ME, Dulucq JL (1994). Complications and recurrences associated with laparoscopic repair of groin hernias A multi-institutional retrospective analysis. Surg Endosc.

[23] Zanghi A, Di Vita M, Lo Menzo E (2011). Multicentric evaluation by Verbal Rate Scale and euroqol-5D of early and late post-operative pain after TAPP and TEP procedures with mechanical fixation for bilateral inguinal hernias. Ann Ital Chir.

[24] Ramshaw B, Shuler FW, Jones HB (2001). Laparoscopic inguinal hernia repair: lessons learned after 1224 consecutive cases. Surg Endosc.

[25] Kald A, Anderberg B, Smedh K, Karlsson M (1997). Transperitoneal or totally extraperitoneal approach in laparoscopic hernia repair: results of 491 consecutive herniorrhaphies. Surg Laparosc Endosc.

[26] Ramshaw BJ, Tucker JG, Conner T, Mason EM, Duncan TD, Lucas GW (1996). A comparison of the approaches to laparoscopic herniorraphy. Surg Endosc.

[27] Gong K, Zhang N, Lu Y (2011). Comparison of the open tension-free mesh-plug, transabdominal preperitoneal (TAPP), and totally extraperitoneal (TEP) laparoscopic techniques for primary unilateral inguinal hernia repair: a prospective randomized controlled trial. Surg Endosc.

[28] Lepere M, Benchetrit S, Debaert M (2000). A multicentric comparison of transabdominal versus totally extraperitoneal laparoscopic hernia repair using PARIETEX meshes. J Soc Laparoendosc Surg.

[29] Belyansky I, Tsirline VB, Klima DA, Walters AL, Lincourt AE, Heniford TB (2011). Prospective, comparative study of postoperative quality of life in TEP, TAPP, and modified Lichtenstein repairs. Ann Surg.

[30] Czechowski A, Schafmayer A (2003). TAPP versus TEP: a retrospective analysis 5 years after laparoscopic transperitoneal and total endoscopic extraperitoneal repair in inguinal and femoral hernia. Chirurg.

[31] Bright E, Reddy VM, Wallace D, Garcea G, Dennison AR (2010). The incidence and success of treatment for severe chronic groin pain after open, transabdominal preperitoneal, and totally extraperitoneal hernia repair. World J Surg.

[32] Felix EL, Michas CA, Gonzalez MH (1995). Laparoscopic hernioplasty—TAPP vs TEP. Surg Endosc.

[33] Bansal VK, Misra MC, Babu D (2013). A prospective, randomized comparison of long-term outcomes: chronic groin pain and quality of life following totally extraperitoneal (TEP) and transabdominal preperitoneal (TAPP) laparoscopic inguinal hernia repair. Surg Endosc Other Interv Tech.

[34] Cohen RV, Alvarez G, Roll S (1998). Transabdominal or totally extraperitoneal laparoscopic hernia repair?. Surg Laparosc Endosc.

[35] Bobrzynski A, Budzynski A, Biesiada Z, Kowalczyk M, Lubikowski J, Sienko J (2001). Experience—the key factor in successful laparoscopic total extraperitoneal and transabdominal preperitoneal hernia repair. Hernia.

[36] Khoury N (1995). A comparative study of laparoscopic extraperitoneal and transabdominal preperitoneal herniorrhaphy. J Laparoendosc Surg.

[37] Berney CR (2012). The Endoloop technique for the primary closure of direct inguinal hernia defect during the endoscopic totally extraperitoneal approach. Hernia.

[38] Reddy VM, Sutton CD, Bloxham L, Garcea G, Ubhi SS, Robertson GS (2007). Laparoscopic repair of direct inguinal hernia: a new technique that reduces the development of postoperative seroma. Hernia.

[39] Lange JF, Lange MM, Voropai DA, van Tilburg MW, Pierie JP, Ploeg RJ, Akkersdijk WL (2014). Trans rectus sheath extra-peritoneal procedure (TREPP) for inguinal hernia: the first 1,000 patients. World J Surg.

[40] Baroody M, Bansal V, Maish G (2004). The open preperitoneal approach to recurrent inguinal hernias in high-risk patients. Hernia.

[41] Ceriani V, Faleschini E, Sarli D, Lodi T, Roncaglia O, Bignami P (2006). Femoral hernia repair. Kugel retroperitoneal approach versus plug alloplasty: a prospective study. Hernia.

[42] Kugel R (1999). Minimally invasive, nonlaparoscopic, preperitoneal, and sutureless, inguinal herniorrhaphy. Am J Surg.

[43] Ceriani V, Faleschini E, Bignami P, Lodi T, Roncaglia O, Osio C (2005). Kugel hernia repair: open ‘‘mini-invasive’’ technique. Personal experience on 620 patients. Hernia.

[44] Akkersdijk WL, Andeweg CS, Bökkerink WJ, Lange JF, van Laarhoven CJ, Koning GG (2016). Teaching the transrectus sheath preperitoneal mesh repair: TREPP in 9 steps. Int J Surg.

[45] Karatepe O, Acet E, Altiok M, Adas G, Cak RA, Karahan S (2010). Preperitoneal repair (open posterior approach) for recurrent inguinal hernias previously treated with Lichtenstein tension-free hernioplasty. Hippokratia.

[46] Van Nieuwenhove Y, Vansteenkiste F, Vierendeels T, Coenye K (2007). Open, preperitoneal hernia repair with the Kugel patch: a prospective, multicentre study of 450 repairs. Hernia.

[47] Bökkerink WJV, Koning GG, Vriens PWHE, Mollen RMHG, Harker MJR, Noordhof RK, Akkersdijk WL, van Laarhoven CJHM (2021). Open Preperitoneal Inguinal Hernia Repair, TREPP Versus TIPP in a Randomized Clinical Trial. Ann Surg.

[48] Andresen K, Rosenberg J (2017). Open preperitoneal groin hernia repair with mesh: A qualitative systematic review. Am J Surg.

[49] Reinhorn, M (2014) Minimally invasive open preperitoneal inguinal hernia repair. J Med Insight 10.24296/jomi/8

[50] Poulose BK, Roll S, Murphy JW (2016). Design and implementation of the Americas Hernia Society Quality Collaborative (AHSQC): improving value in hernia care. Hernia.

[51] Filip E. Muysoms, Aude Vanlander, Robrecht Ceulemans, Iris Kyle-Leinhase, Maarten Michiels, Ivo Jacobs, Pieter Pletinckx, Frederik Berrevoet (2016) A prospective, multicenter, observational study on quality of life after laparoscopic inguinal hernia repair with ProGrip laparoscopic, self-fixating mesh according to the European Registry for Abdominal Wall Hernias Quality of Life Instrument. Surgery 160(5): 1344–1357 10.1016/j.surg.2016.04.026 27316825

[52] Hosmer DW, Lemeshow S (1999). Applied Survival Analysis.

[53] George B, Seals S, Aban I (2014). Survival analysis and regression models. J Nucl Cardiol.

[54] Luijendijk RW, Hop WC, van den Tol MP (2000). A comparison of suture repair with mesh repair for incisional hernia. N Engl J Med.

[55] Burger JW, Luijendijk RW, Hop WC, Halm JA, Verdaasdonk EG, Jeekel J (2004). Long-term follow-up of a randomized controlled trial of suture versus mesh repair of incisional hernia. Ann Surg.

[56] Austin PC (2011). An introduction to propensity score methods for reducing the effects of confounding in observational studies. Multivar Behav Res.

[57] Austin PC (2010). Statistical criteria for selecting the optimal number of untreated subjects matched to each treated subject when using many-to-one matching on the propensity score. Am J Epidemiol.

[58] Mitra R, Reiter JP (2016). A comparison of two methods of estimating propensity scores after multiple imputation. Stat Methods Med Res.

[59] Nordin P, Zetterström H, Gunnarsson U, Nilsson E (2003). Local, regional, or general anaesthesia in groin hernia repair: multicentre randomised trial. Lancet.

[60] Nordin P, Zetterström H, Carlsson P, Nilsson E (2007). Cost-effectiveness analysis of local, regional and general anaesthesia for inguinal hernia repair using data from a randomized clinical trial. Br J Surg.

[61] Bender O, Balcı FL, Yüney E, Sağlam F, Ozdenkaya Y, Sarı YS (2009). Systemic inflammatory response after Kugel versus laparoscopic groin hernia repair: a prospective randomized trial. Surg Endosc.

[62] Schwab R, Eissele S, Brückner U, Gebhard F, Becker H (2004). Systemic inflammatory response after endoscopic (TEP) vs Shouldice groin hernia repair. Hernia.

[63] McCormack K, Scott NW, Go PM, Ross S, Grant AM (2003) Laparoscopic techniques versus open techniques for inguinal hernia repair. Cochrane Database Syst Rev (1):CD001785. 10.1002/14651858.CD001785 10.1002/14651858.CD001785 PMC8407507 12535413

[64] Schmedt CG, Sauerland S, Bittner R (2005). Comparison of endoscopic procedures vs Lichtenstein and other open mesh techniques for inguinal hernia repair: a meta-analysis of randomized controlled trials. Surg Endosc.

[65] Bittner R, Sauerland S, Schmedt C-G (2005). Comparison of endoscopic techniques vs Shouldice and other open nonmesh techniques for inguinal hernia repair: a meta-analysis of randomized controlled trials. Surg Endosc.

[66] Schmedt CG, Leibl BJ, Bittner R (2002). Endoscopic inguinal hernia repair in comparison with Shouldice and Lichtenstein repair. A systematic review of randomized trials. Dig Surg.

[67] Mylonas KS, Reinhorn M, Ott LR, Westfal ML, Masiakos PT (2017). Patient-reported opioid analgesic requirements after elective inguinal hernia repair: A call for procedure-specific opioid-administration strategies. Surgery.

[68] Linnaus ME, Sheaffer WW, Ali-Mucheru MN, Velazco CS, Neville M, Gray RJ (2019). The opioid crisis and surgeons: national survey of prescribing patterns and the influence of motivators, experience, and gender. Am J Surg.

[69] Higgins RM, Petro CC, Warren J (2022). The opioid reduction task force: using the ACHQC Data Registry to combat an epidemic in hernia patients. Hernia.

[70] Antonescu I, Baldini G, Watson D (2013). Impact of a bladder scan protocol on discharge efficiency within a care pathway for ambulatory inguinal herniorrhaphy. Surg Endosc.

[71] Koch CA, Grinberg GG, Farley DR (2006). Incidence and risk factors for urinary retention after endoscopic hernia repair. Am J Surg.

[72] Jensen P, Mikkelsen T, Kehlet H (2002). Postherniorrhaphy urinary retention—effect of local, regional, and general anesthesia: a review. Reg Anesth Pain Med.

[73] Treadwell J, Tipton K, Oyesanmi O, Sun F, Schoelles K (2012). Surgical options for inguinal hernia: comparative effectiveness review. Agency Healthc Res Qual Comp.

[74] Ericsson A, Pool R (2017). *Peak: secrets from the new science of expertise.* First Mariner.

