# Supplementary material for: Posterior mesh inguinal hernia repairs: a propensity score matched analysis of laparoscopic and robotic versus open approaches
Source: Hernia. 2022 Sep 20;27(1):93–104. doi: 10.1007/s10029-022-02680-0 (PMC9931785; doi:10.1007/s10029-022-02680-0)

Supplemental Tables

Supplemental Table 1: TAPP vs TREPP Subgroup Analysis

|  | TAPP | TREPP | P-value | OR | 95% CI |
| --- | --- | --- | --- | --- | --- |
| Number of matched pairs | N=611 | N=611 |  |  |  |
| EuraHS QOL 30d  Median (IQR) | 9.00 (3.00-22.0) | 7.00 (2.00-17.61) | 0.018 | 0.731 | (0.563, 0.947) |
| N | 336 | 355 |  |  |  |
| EuraHS QOL 180d Median (IQR) | 2.00 (0.00-6.00) | 1.00 (0.00-4.00) | 0.122 | 0.756 | (0.53, 1.078) |
| N | 177 | 225 |  |  |  |
| EuraHS QOL 1 year Median (IQR) | 1.00 (0.00-4.33) | 1.00 (0.00-4.00) | 0.49 | 0.86 | (0.52, 1.362) |
| N | 101 | 127 |  |  |  |
| EuraHS pain 30d (post-hoc) Median (IQR) | 3.00 (0.00-6.00) | 2.00 (0.00-4.00) | - | - | - |
| N | 336 | 355 |  |  |  |
| EuraHS pain 180d (post-hoc) Median (IQR) | 0.00 (0.00-2.00) | 0.00 (0.00-1.00) | - | - | - |
| N | 177 | 225 |  |  |  |

Supplemental Table 2: TEP vs TREPP Subgroup Analysis

|  | TEP | TREPP | P-value | OR | 95% CI |
| --- | --- | --- | --- | --- | --- |
| Number of matched pairs | N=714 | N=714 |  |  |  |
| EuraHS QOL 30d  Median (IQR) | 8.00 (3.00-22.00) | 7.00 (2.00-16.86) | 0.043 | 0.772 | (0.601, .0992) |
| N | 390 | 352 |  |  |  |
| EuraHS QOL 180d  Median (IQR) | 1.00 (0.00-6.58) | 1.00 (0.00-4.08) | 0.593 | 0.901 | (0.614, 1.322) |
| N | 140 | 238 |  |  |  |
| EuraHS QOL 1 year  Median (IQR) | 0.00 (0.00-4.00) | 0.50 (0.00-4.00) | 0.813 | 1.064 | (0.693, 1.783) |
| N | 76 | 154 |  |  |  |
| EuraHS pain 30d (post-hoc) Median (IQR) | 2.00 (0.00-6.00) | 2.00 (0.00-4.00) | - | - | - |
| N | 390 | 352 |  |  |  |
| EuraHS pain 180d (post-hoc) Median (IQR) | 0.00 (0.00-1.58) | 0.00 (0.00-0.08) | - | - | - |
| N | 140 | 238 |  |  |  |

Supplemental Table 3: rTAPP vs TREPP Subgroup Analysis

|  | rTAPP | TREPP | P-value | OR | 95% CI |
| --- | --- | --- | --- | --- | --- |
|  | N=705 | N=705 |  |  |  |
| EuraHS QOL 30d  Median (IQR) | 11.00 (3.00-27.00) | 8.00 (2.00-17.69) | 0.002 | 0.684 | (0.535, 0.872) |
| N | 368 | 422 |  |  |  |
| EuraHS QOL 180d  Median (IQR) | 2.00 (0.00-7.00) | 1.00 (0.00-5.00) | 0.225 | 0.813 | (0.581, 1.137) |
| N | 191 | 276 |  |  |  |
| EuraHS QOL 1 year  Median (IQR) | 1.50 (0.00-6.00) | 1.00 (0.00-4.00) | 0.116 | 0.708 | (0.46, 1.089) |
| N | 116 | 172 |  |  |  |
| EuraHS pain 30d (post-hoc) Median (IQR) | 3.00 (0.00-6.58) | 2.00 (0.00-4.00) | - | - | - |
| N | 368 | 422 |  |  |  |
| EuraHS pain 180d (post-hoc) Median (IQR) | 0.00 (0.00-1.83) | 0.00 (0.00-1.00) | - | - | - |
| N | 191 | 276 |  |  |  |

Supplemental Figure 1. Recurrence-free probability of TAPP, TEP and rTAPP vs TREPP repairs


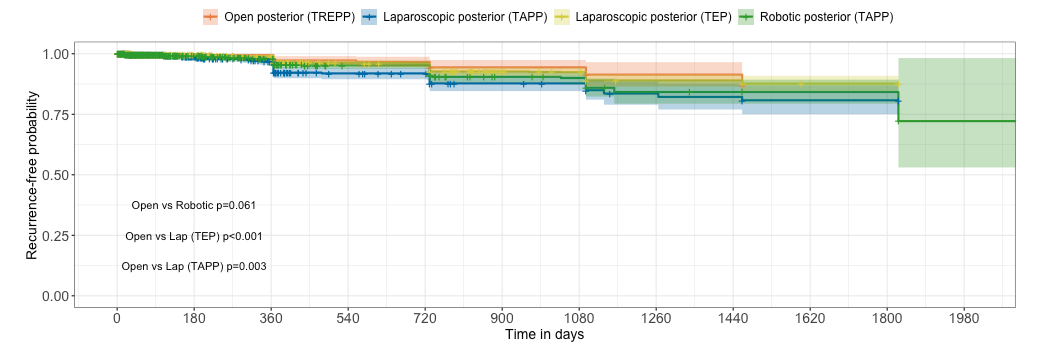

Supplement: Supplementary file 1 — Supplementary file1 (DOCX 63 KB) [file 10029_2022_2680_MOESM1_ESM.docx]
